# LW-FQZip 2: a parallelized reference-based compression of FASTQ files

**DOI:** 10.1186/s12859-017-1588-x

**Published:** 2017-03-20

**Authors:** Zhi-An Huang, Zhenkun Wen, Qingjin Deng, Ying Chu, Yiwen Sun, Zexuan Zhu

**Affiliations:** 1 College of Computer Science and Software Engineering, Shenzhen University, Shenzhen, 518060 China; 2 School of Medicine, Shenzhen University, Shenzhen, 518060 China

**Keywords:** High-throughput sequencing, Sequencing data compression, Reference- based compression, Sequence alignment

## Abstract

**Background:**

The rapid progress of high-throughput DNA sequencing techniques has dramatically reduced the costs of whole genome sequencing, which leads to revolutionary advances in gene industry. The explosively increasing volume of raw data outpaces the decreasing disk cost and the storage of huge sequencing data has become a bottleneck of downstream analyses. Data compression is considered as a solution to reduce the dependency on storage. Efficient sequencing data compression methods are highly demanded.

**Results:**

In this article, we present a lossless reference-based compression method namely LW-FQZip 2 targeted at FASTQ files. LW-FQZip 2 is improved from LW-FQZip 1 by introducing more efficient coding scheme and parallelism. Particularly, LW-FQZip 2 is equipped with a light-weight mapping model, bitwise prediction by partial matching model, arithmetic coding, and multi-threading parallelism. LW-FQZip 2 is evaluated on both short-read and long-read data generated from various sequencing platforms. The experimental results show that LW-FQZip 2 is able to obtain promising compression ratios at reasonable time and memory space costs.

**Conclusions:**

The competence enables LW-FQZip 2 to serve as a candidate tool for archival or space-sensitive applications of high-throughput DNA sequencing data. LW-FQZip 2 is freely available at http://csse.szu.edu.cn/staff/zhuzx/LWFQZip2 and https://github.com/Zhuzxlab/LW-FQZip2.

**Electronic supplementary material:**

The online version of this article (doi:10.1186/s12859-017-1588-x) contains supplementary material, which is available to authorized users.

## Background

The rapid progress of high-throughput DNA sequencing techniques has dramatically reduced the costs of whole genome sequencing, which leads to revolutionary advances in gene industry [1, 2]. Genome studies have produced tremendous volume of data that poses great challenges to storage and transfer. Data compression becomes necessary as a silver-bullet solution to ease the dilemma [3–6]. Nevertheless, the popular generic compression tools, such as gzip (http://www.gzip.org/) and bzip2 (http://www.bzip.org), cannot obtain satisfactory performance on high-throughput DNA sequencing data, because they do not utilize the biological characteristics of the data like repeat fragments and palindromes. Efficient compression methods oriented to high-throughput DNA sequencing data are highly demanded.

Many specialized compression methods have been proposed for sequencing data in raw FASTQ format [7–11], sequencing reads (DNA nucleotides only) [12–15] and/or aligned SAM/BAM format [16]. Depending on whether extra reference genomes are required, these compression methods normally can be classified into reference-based and reference-free methods.

Reference-based methods align the targeted sequences to some external reference sequence(s) for identifying the similarity between them. The variances of the alignment are encoded instead of the original targeted sequences. Reference-based methods generally obtain better compression ratios with more time consumption by involving a sequence alignment pre-processing. Representative state-of-the-art reference-based methods include CRAM [17], Quip [11], and DeeZ [18]. CRAM [17], working with BAM-based input, records the variances of reads and a reference genome with Huffman coding. Quip [11] with ‘-r’ option compresses SAM/BAM data in a standard reference-based scheme and employs highly optimized statistical models for various SAM fields, thus leading to compromising compression rate. It is also applicable to reference-based FASTQ compression in ‘-a’ mode where a *de novo* assembly procedure is introduced to construct references in the target data rather than using existing references. DeeZ [18] lowers the cost of representing common differences among the reads’ mapping results by implicitly assembling the underlying donor genome in order to encode these variants only once.

Reference-free methods compress the raw sequencing data, mainly in FASTQ format, directly based on their natural statistics. For example, FQZcomp [8] uses an order-k context model to predict the nucleotide sequences in FASTQ format followed by arithmetic coding based compression. DSRC [10] partitions input FASTQ data into blocks enabling independent compression of them using LZ77 and Huffman encoding schemes. DSRC 2 [19] is an improvement of DSRC by introducing threaded parallelism and more efficient coding scheme. LFQC [20] preforms data transformation to the four fields of the sequencing records in an FASTQ file separately, followed by regular data compressor namely zpaq and lpaq8. LEON [21] first builds a reference as a probabilistic *de Bruijn graph* based on bloom filter, and then records the reads and quality scores as mapped paths in the graph using arithmetic encoding. SCALCE [22] reorganizes reads in an FASTQ file that share common ‘core’ substrings into groups, and then compacts the groups using gzip or LZ77-like tools. SeqDB [23] coordinates sequences bases and their corresponding quality scores into 2D byte arrays and compresses them with an existing multithreaded compressor Blosc. Quip [11], in addition to the reference-based compression mode, also provides reference-free compression using arithmetic coding based on high order Markov chains. Instead of exploiting the redundancy of homologous sequences, reference-free methods put more effort into predictive model and coding scheme, which tends to improve the time efficiency by sacrificing compression ratio to some degree [24].

This work focus on long-term archiving and space-sensitive scenarios, where superior compression ratio is pursued and reference-based methods are more favourable. In our previous work [25], we have proposed a self-contained reference-based method, namely LW-FQZip 1, to compress high-throughput DNA sequencing data in raw FASTQ format. LW-FQZip 1 introduces a light-weight mapping model to efficiently align short reads against the reference sequence based on a *k*-mer indexing strategy. The light-weight mapping model distinguishes LW-FQZip 1 from other reference-based methods for not relying on any external alignment software. Nevertheless, LW-FQZip 1 is far from satisfactory in terms of compression efficiency.

LW-FQZip 2 is an improved version of LW-FQZip 1 by introducing parallelism and more efficient coding schemes. Especially, LW-FQZip 2 is equipped with light-weight mapping model, bitwise prediction by partial matching (PPM), arithmetic coding, and multi-threading parallelism. It can support various FASTQ files generated from the most well-known high-throughput sequencing platforms and obtain superior compression ratios at reasonable time and memory space costs.

## Implementation

**Fig. 1:**
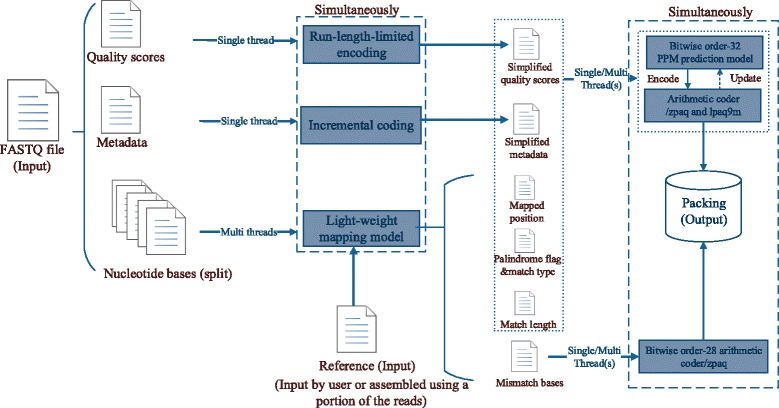
The general framework of LW-FQZip 2. Firstly, the input FASTQ file is split into three data streams of metadata, bases, and quality scores. Secondly, the quality scores and metadata are compacted with run-length-limited encoding and incremental encoding, respectively. The nucleotide bases are partitioned and mapped to an external reference sequence based on the light-weight mapping model. Finally, the processed intermediate files from the three streams are compressed with arithmetic coder and/or other specific coding schemes

The general framework of LW-FQZip 2 is shown in Fig. 1. Firstly, LW-FQZip 2 splits an input FASTQ file into three data streams (i.e., metadata, nucleotide sequences, and quality scores) and then the nucleotide sequences (also known as reads) are divided into equal-sized sub-blocks which are simultaneously fed to the light-weight mapping model implemented with multi-threading. After the sequence mapping, the matching results (i.e., the mapped position, palindrome flag, match length, and match type) and mismatch values are recorded in different intermediate files. Secondly, the metadata and quality scores are simultaneously proceeded by abridging the consecutive repeats with incremental encoding and run-length-limited encoding, respectively. Finally, the intermediate files generated from the metadata, nucleotide sequences, and quality scores streams are compacted with a combination of bitwise order-32 PPM model and arithmetic coder in parallel, except that the intermediate file storing mismatch values is compressed by a distinct bitwise order-28 arithmetic coder. In best compression ratio mode, i.e., LW-FQZip 2 with a ‘–g’ option selected, the quality scores and mismatch values are compacted using the zpaq tool (http://mattmahoney.net/dc/zpaq.html) and the other intermediate files encoded with lpaq9m (http://mattmahoney.net/dc/text.html#1440). LW-FQZip 2 is available at http://csse.szu.edu.cn/staff/zhuzx/LWFQZip2 and https://github.com/Zhuzxlab/LW-FQZip2. The pseudo-code of LW-FQZip 2 is provided in Algorithm 1, Additional file 1. The key components of LW-FQZip 2 are described as follows.

### Compression of metadata and quality scores

In LW-FQZip 2, the metadata are pre-processed with incremental encoding, with which the variances of one metadata to its previous neighbour is stored rather than the original data. The quality scores are pre-processed with run-length-limited encoding. More details of the incremental encoding and run-length-limited encoding are available in [25].

After pre-processing, the processed data can be compressed with lpaq9m (if ‘-g’ option is selected) or a combination of PPM model and arithmetic coder. In the latter case, a binary tree is established to store the predictive context information. The pre-processed metadata and quality scores are transformed into binary streams to train an order-32 PPM model, i.e., prediction based on a 4-byte context (higher order can improve the compression ratios by promoting the predictive accuracy but it consumes more memory space and running time. A trade-off order 32 is adopted in this study). The binary quality scores are matched against the predicted results per bit by producing ‘0’ or ‘1’, and then the context prediction model is updated accordingly. Finally, the prediction results are recorded using arithmetic coder or zpaq (if ‘-g’ option is selected). The pseudo-code of the compression with PPM prediction model and arithmetic coding/zpaq is provided in Algorithm 2, Additional file 1.

### Reference-based compression of nucleotide sequences using light-weight mapping model

The target nucleotide sequences are mapped to an external reference sequence based on the light-weight mapping model [25] and the mapping results are recorded instead of the original sequences.

To make this article self-contained, the light-weight mapping model is briefly introduced in this subsection. The mapping model is designed to implement fast alignment by indexing the *k*-mer substrings within the reference. A hash table ***I***_***R***_ is firstly established to save all positions of *k*-mer substrings in the reference with some predefined prefix, e.g., ‘CG’. On mapping a read of nucleotide bases ***X*** to the reference, the model identifies all *k*-mer substring included in ***I***_***R***_ and selects the valuable *k*-mer substrings served as seeds, where some restrictions are predefined to eliminate the low-quality *k*-mer substrings (e.g., minimum seed length ***L***, mismatch tolerance rate ***e***). Based on the selected seeds, multiple local alignments are performed to identify the maximum matches. The mapping results of ***X*** including the mapped position, palindrome flag, match length, match type and mismatch values are recorded in some intermediate files. If no *k*-mer substring in ***X*** is identified in ***I***_***R***_, the palindrome of ***X*** undergoes the same mapping procedure. In the case neither ***X*** nor its palindrome is mapped to the reference, the plain ***X*** is output for encoding directly.

To improve the matching rate, the unmapped parts of reads are further partitioned into shorter segments and realigned against the reference where palindrome match is considered. The mismatched nucleotide bases are composed exclusively of four characters (i.e., {‘A’, ‘C’, ‘G’, ‘T’}), which are much easier to encode than quality scores. Therefore, a simpler yet efficient model based on the bitwise order-28 arithmetic coder (http://cs.fit.edu/~mmahoney/compression/text.html#2212) or zpaq is adopted. If no proper reference is available, LW-FQZip 2 also provides an option ‘-a’ to generate a reference by assembling a portion of reads that contain the predefined prefix.

### Blocking and multithreading

Parallelism is introduced to LW-FQZip 2 to improve the computational efficiency using the Pthreads library. In the mapping procedure, the input FASTQ file is partitioned into ***b*** (empirically set to 10 in this study) equal-sized blocks. Accordingly, ***b*** threads are simultaneously executed with each running a light-weight mapping model for a corresponding block. Afterward, a new single thread is created to collect the mapping results of the previous ***b*** threads and dispatch the results into different intermediate files. After the three data streams, i.e., metadata, quality scores, and nucleotide sequences are properly processed, multiple threads are created to compress the six intermediate files with the corresponding encoding schemes as shown in Fig. 1.

In summary, LW-FQZip 2 improves LW-FQZip 1 by introducing multi-threading for the time-consuming read mapping and using more efficient encoding schemes based on PPM model, arithmetic coders, lpaq9m and/or zpaq. The implementation details of LW-FQZip 2 are provided in the Additional file 1.

## Results and discussion

LW-FQZip 2 is verified using ten representative real-world FASTQ files (five short-read data and five long-read data) on a platform running 64-bit Red Hat 4.4.7-16 with four 8-core Intel(R) Xeon(R) E7-8837 CPUs (@2.67GHz with Hyper-Threading Technology). These data sets, generated from various well-known high-throughput DNA sequencing platforms, were downloaded from the Sequence Read Archive of the National Centre for Biotechnology Information (NCBI) [26]. Details of these data sets are provided in Table 1.

**Table 1:** The ten real-world FASTQ data sets used for performance evaluation

	Datasets	Platforms	Species	Read length (bp)	Size (MB)	GC content
Long-read	SRR2916693	454GS	Pseudomonas moraviensis	67-1201	425	58.8%
	SRR2994368	Illumina Miseq	Escherichia coli	70-502	4688	49.7%
	SRR3211986	Pacbio RS	Homo sapiens	2-62746	1759	39.6%
	ERR739513	MinION	Phage	5-246140	871	47.9%
	SRR3190692	Illumina MiSeq	Escherichia coli	70-602	11379	52.3%
Short-read	ERR385912	Illumina Hiseq 2000	Escherichia coli	51	641	43.5%
	ERR386131	Ion Torrent PGM	Capsicum baccatum	151	1371	50.5%
	SRR034509	Illumina Analyzer II	Escherichia coli	101	5247	52.6%
	ERR174310	Illumina Hiseq 2000	Homo sapiens	202	105122	N.A.
	ERR194147	Illumina Hiseq 2000	Homo sapiens	101	202631	40.3%

*Note*: The long-read data sets have variable-length reads, while the short-read data sets have fixed-length reads

LW-FQZip 2 is compared with the other state-of-the-art lossless FASTQ compressors namely Quip [11], DSRC 2 [19], FQZcomp [8], CRAM [17], LFQC [20], LEON [21] and SCALCE [22]. The general-purpose compression tools, i.e., gzip and bzip2, as well as the original LW-FQZip 1 are also included in the comparison as baselines. All compared methods are configured to obtain the best compression ratio (the parameter settings of all methods and the software version information are provided in the Additional file 1). LW-FQZip 2 is evaluated on two modes, i.e., the normal mode (‘LW-FQZip 2’, using arithmetic coders to compress the intermediate files) and the best compression ratio mode (‘LW-FQZip 2 (−g)’, using lpaq9m and zpaq to compress the intermediate files). Quip is also executed in two modes, i.e., the reference-based compression (‘quip -r’) and the assembly-based compression (‘quip -a’, a reference is assembled with a portion of reads, and then a reference-based compression is conducted using the generated reference). Quip, CRAM, and LW-FQZip 1 fall within reference-based scheme. The other methods are reference-free methods.

**Fig. 2:**
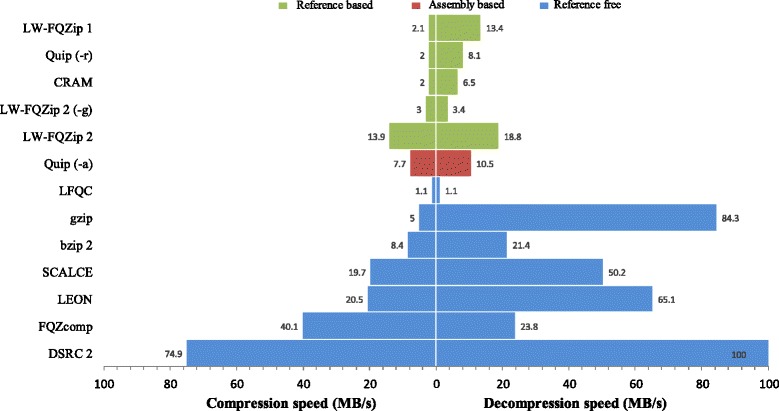
The average compression and decompression speeds of the compared methods on ten test data sets. The compression speed is calculated as the original file size divided by the compression time. The decompression speed is calculated as the original file size divided by the decompression time

The performance of the methods is evaluated in terms of compression ratio, speed, and memory consumption. The compression ratios of all compared methods on the ten FASTQ data sets are tabulated in Table 2. The average compression and decompression speeds of all methods are plotted in Fig. 2. The memory sizes consumed by the compared methods on each data set are reported in Table 3. More details of the data sets and experimental studies are provided in Additional file 1: Tables S1-S13. The average CPU utilization and version information of all compared methods are provided in Additional file 1: Tables S14 and S17, respectively.

**Table 2:** The compression ratios of the compared methods on ten test data sets

		LW-FQZip 2	LW-FQZip 2 (−g)	LW-FQZip 1	Quip (−a)	Quip (−r)	DSRC 2	CRAM	FQZcomp	LFQC	LEON	SCALCE	bzip 2	gzip
Long-read	SRR2916693	16.7%	15.3%	18.1%	20.9%	20.5%	20.2%	21.9%	21.6%	**12.7%**	19.5%	17.2%^a^	24.2%	29.6%
	SRR2994368	17.3%	**16.0%**	17.9%	20.1%	N/A	23.2%	26.4%	N/A	N/A	23.1%	17.3%^a^	28.5%	34.2%
	SRR3211986	33.3%	**32.3%**	N/A	33.3%	N/A	N/A	33.9%	N/A	**32.3%**	N/A	33.4%^a^	36.4%	42.6%
	ERR739513	35.2%	**34.8%**	N/A	N/A	N/A	N/A	35.6%	N/A	34.9%	N/A	N/A	39.7%	45.4%
	SRR3190692	12.7%	**11.7%**	13.2%	16.5%	N/A	20.3%	22.3%	N/A	N/A	18.1%	12.7%^a^	24.4%	29.5%
Short-read	ERR385912	6.4%	**5.0%**	6.6%	7.2%	N/A	7.8%	N/A	N/A	5.8%	7.0%	6.6%^a^	13.9%	17.9%
	ERR386131	16.5%	16.0%	18.7%	17.7%	16.6%	16.8%	25.5%	24.6%	**15.5%**	N/A	16.6%^a^	21.5%	26.0%
	SRR034509	23.7%	**22.7%**	25.0%	25.1%	24.9%	26.1%	27.4%	26.1%	23.7%	27.9%	24.5%^a^	31.5%	36.9%
	ERR174310	21.0%	20.1%	N/A	**20.0%**	N/A	20.2%	N/A	N/A	N/A	25.3%	19.6%^a^	26.2%	31.7%
	ERR194147	20.1%	**14.3%**	N/A	20.0%	N/A	20.3%	N/A	N/A	N/A	20.3%	15.4%^a^	19.7%	23.6%

Compressed Ratio: the compressed file size divided by the original file size; ‘N/A’: the program cannot work on the data, some error occur in program, such as loses fidelity after decompression or decompression failed; ‘^a^’: the read order is changed after decompression; The best results are highlighted in bold

**Table 3:** The memory usage (MB) of the compared methods on ten test data sets

		LW- FQZip 2	LW- FQZip 2 (−g)	LW- FQZip 1	Quip (−a)	Quip (−r)	DSRC 2	CRAM	FQZcomp	LFQC	SCALCE	LEON	bzip 2	gzip
SRR2916693	compression	1605	4420	459	759	391	582	784	312	3965	1380	1722	7.6	**1.6**
	decompression	1598	4127	37	756	389	601	620	314	3932	1050	696	4.8	**1.5**
SRR2994368	compression	1582	14158	1048	2801	389	6175	1355	N/A	N/A	4073	5435	7.6	**1.6**
	decompression	1579	13154	69	2198	387	7234	652	N/A	N/A	1050	2623	4.8	**1.5**
SRR3211986	compression	1190	12935	N/A	1098	N/A	N/A	5777	N/A	4768	2158	N/A	7.6	**1.6**
	decompression	1528	5657	N/A	1109	N/A	N/A	2381	N/A	4320	1035	N/A	4.8	**1.5**
ERR739513	compression	1283	11511	N/A	N/A	N/A	N/A	3694	N/A	5108	N/A	N/A	7.6	**1.6**
	decompression	1403	11079	N/A	N/A	N/A	N/A	1455	N/A	4748	N/A	N/A	4.8	**1.5**
SRR3190692	compression	1726	14560	1058	3552	391	14157	1363	N/A	N/A	5219	6776	7.6	**1.6**
	decompression	1725	13329	69	2898	386	14794	661	N/A	N/A	1055	3217	4.8	**1.5**
ERR385912	compression	1603	2793	410	772	389	911	N/A	N/A	3140	1422	1717	7.6	**1.6**
	decompression	1603	2655	69	770	392	908	N/A	N/A	3060	1040	504	4.8	**1.5**
ERR386131	compression	1691	12443	1033	771	389	1844	1318	322	5175	1961	N/A	7.6	**1.6**
	decompression	1721	12165	39	768	384	1950	651	319	4835	1049	N/A	4.8	**1.5**
SRR034509	compression	1748	14670	1073	1531	383	6683	1351	324	5352	4151	4139	7.6	**1.6**
	decompression	1752	7042	71	1218	391	7736	653	309	4859	1050	1799	4.8	**1.5**
ERR174310	compression	1886	16270	N/A	5333	N/A	5558	N/A	N/A	N/A	5333	7797	7.6	**1.6**
	decompression	1865	11239	N/A	1156	N/A	18487	N/A	N/A	N/A	1045	5106	4.8	**1.5**
ERR194147	compression	1953	17771	N/A	782	N/A	20271	N/A	N/A	N/A	5380	7192	7.6	**1.6**
	decompression	1963	13908	N/A	780	N/A	24284	N/A	N/A	N/A	1057	5322	4.8	**1.5**

*Note*: The best results are highlighted in bold

**Fig. 3:**
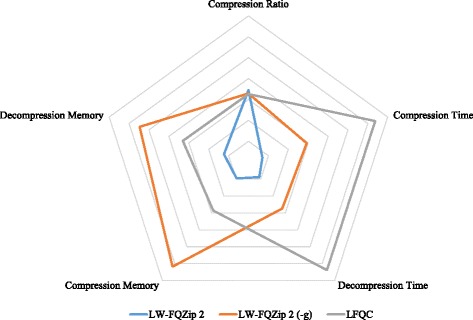
Comparison between LW-FQZip 2, LW-FQZip 2 (−g) and LFQC in a radar chart in terms of average compression ratio, compression time, decompression time, compression memory usage, and decompression usage. In each criterion, the results of the three methods are normalized to the range of [0, 1] and a smaller value, i.e., closer to the centroid, indicates a better performance

From Table 2, it is shown that LW-FQZip 2 and LW-FQZip 2 (−g) successfully work on all test data sets. LW-FQZip 2 (−g) tends to obtain superior compression ratios to the other methods especially on long-read data. DSRC 2, Quip, FQZcomp, LEON, SCALCE and LW-FQZip 1 suffer from some issues like incompatibility and fidelity-loss on the long-read data generated from Pacbio RS and MinION platforms. LW-FQZip 1, CRAM, FQZcomp and LFQC also fail on some short-read data sets of large size. LFQC obtains comparable compression ratios to LW-FQZip 2 and LW-FQZip 2 (−g) in the data sets it works out. We made an extra comparison analysis between LW-FQZip 2, LW-FQZip (−g) and LFQC in terms of compression ratio, memory usage, and time consumption in a radar chart in Fig. 3. The results show that LW-FQZip (−g) and LFQC attain slightly better compression ratios than LW-FQZip 2 at the cost of memory usage and time consumption, respectively. Nevertheless, LW-FQZip 2 achieves better compromise over all metrics than the other two methods.

In terms of compression and decompression speeds, LW-FQZip 2 outperforms other reference-based methods as shown in Fig. 2. It is worth highlighting that LW-FQZip 2 outperforms LW-FQZip 1 in terms of compatibility, compression ratio, and speed, which suggests a substantial improvement of LW-FQZip 2 to LW-FQZip 1. As expected, reference-free methods tend to be faster than reference-based methods. Among the reference-free methods, DSRC 2 compresses the fastest with compromising compression ratio by taking full advantage of multithreading. Quip and LEON manage to obtain some trade-offs between the three evaluation criteria. SCALCE is a very efficient tool by introducing a boosting scheme based on locally consistent parsing (LCP) technique to sort the reads, which enables SCALCE to compress similar reads together and attain competitive compression ratios at high speed.

We also try to adopt the LCP technique in our method as the framework shown in Additional file 1: Figure S2. In this attempt, the successfully mapped reads are still compressed with the original LW-FQZip 2, whereas the unmapped reads undergo the LCP boosting and gzip compression. LCP is applied to only a small portion of the reads, yet the results shown in Additional file 1: Table S21, suggest that LCP can improve the compression ratio. Indeed, re-sorting the reads according to their similarity is really an efficient option to improve the compression ratio, especially for archiving-oriented applications. However, since the order of the reads is changed, it inevitably imposes extra cost if random access of the archive is the concern in the downstream analysis. LW-FQZip 2 is designed to preserve the original read order to facilitate the implementation of random access in the future extension of this tool.

The compared methods are also tested on the benchmark data sets suggested by the MPEG working group on genomic compression (https://github.com/sfu-compbio/compression-benchmark/blob/master/samples.md). The results are presented in Additional file 1: Tables S18-S20, where the proposed method shows consistent efficiency.

The experimental results suggest that all specialized methods outperform the general-purpose tools in terms of compression ratio but use more memory space. The reference-based methods tend to be slower than the reference-free methods, due to the extra running time involved in the sequence alignment preprocessing. Different methods are designed with different strengths and can be used for different purposes. There is no single method dominates other methods in all criteria.

The effect of palindrome handling is investigated in the Additional file 1: Tables S15 and S16. With palindrome handling more reads can be mapped to the reference at a higher speed (the mapping is stopped once the first match is identified), thus the compression ratio and speed are improved slightly, while the extra memory consumption is eligible.

The effect of thread number is studied in Additional file 1: Figure S1. The compression speed is affected not only by the number of threads but also the disk I/O speed. As a result, the compression might not be speeded up proportionally as the number of threads increases (as shown in Additional file 1: Figure S1). Using faster disk system like solid state disk (SSD) can help to speed up both compression and decompression (see in the Additional file 1: Table S22).

## Conclusions

This article presents a specialized compression tool LW-FQZip 2 for FASTQ files. LW-FQZip 2 shows superior compression ratios and compatibility with reasonable (de) compression speed and memory space consumption. It could serve as a candidate tool for archival or storage space-sensitive applications of sequencing data.

The emerging long-read technologies, e.g., Single Molecule Real Time (SMRT) sequencing [27] and Nanopore sequencing [28], produce much longer DNA sequences, reportedly providing a more complete picture of genome structure. They are deemed to be a complementary solution to overcome the shortages of short-read sequencing. The exponentially increasing long-read sequencing data poses new great challenges to the existing specialized compression methods. LW-FQZip 2 shows good compatibility to long-read sequencing data. The current work is hoped to provide insights into the storage problems of new sequencing data. More efficient alignment models for long-read data will be developed in the future work.

## Additional file

Additional file 1: This document provides the implementation details of LW-FQZip 2 and the detailed experimental results of each compared algorithm in the related comparison study. **Algorithm 1.** The main procedure of LW-FQZip 2. **Algorithm 2.** Compression with PPM prediction model and arithmetic coding. **Table S1.** The performance of LW-FQZip 2. **Table S2.** The performance of LW-FQZip 2 (−g). **Table S3.** The performance of LW-FQZip 1. **Table S4.** The performance of Quip (−a). **Table S5.** The performance of Quip (−r). **Table S6.** The performance of DSRC 2. **Table S7.** The performance of CRAM. **Table S8.** The performance of FQZcomp. **Table S9.** The performance of LFQC. **Table S10.** The performance of LEON. **Table S11.** The performance of SCALCE. **Table S12.** The performance of bzip 2. **Table S13.** The performance of gzip. **Table S14.** The average number of CPU cores used by the compared methods. **Table S15.** The compression ratios and time consumptions of LW-FQZip 2 w/o complementary palindrome mapping. **Table S16.** The memory usage of the LW-FQZip 2 w/o complementary palindrome mapping. **Table S17.** The version information of all compared methods. **Table S18.** The compression ratios of the compared methods on benchmark data provided by MPEG working group on genomic compression. **Table S19.** The performance of LW-FQZip 2 on benchmark data provided by MPEG working group on genomic compression. **Table S20.** The performance of LW-FQZip 2 (−g) on benchmark data provided by MPEG working group on genomic compression. **Table S21.** The compression ratios of LW-FQZip2 + LCP, LW-FQZip2 -g + LCP, SCALCE, and LW-FQZip 2 on seven representative data sets. **Table S22.** The comparison of compression speed of LW-FQZip 2 using SSD and HDD disk systems. **Figure S1.** The compression speeds of LW-FQZip 2 using different number of threads on five representative data sets. **Figure S2.** The framework of LW-FQZip 2 with LCP technique. (DOCX 229 kb)

## Abbreviations

PPM: Prediction by partial matching
LCP: Locally consistent parsing
NCBI: National Centre for Biotechnology Information
SMRT: Single molecule real time
SSD: Solid state disk
