# Supplementary material for: LW-FQZip 2: a parallelized reference-based compression of FASTQ files
Source: BMC Bioinformatics. 2017 Mar 20;18:179. doi: 10.1186/s12859-017-1588-x (PMC5359991; doi:10.1186/s12859-017-1588-x)
Supplement: Additional file 1: — This document provides the implementation details of LW-FQZip 2 and the detailed experimental results of each compared algorithm in the related comparison study. Algorithm 1. The main procedure of LW-FQZip 2. Algorithm 2. Compression with PPM prediction model and arithmetic coding. Table S1. The performance of LW-FQZip 2. Table S2. The performance of LW-FQZip 2 (−g). Table S3. The performance of LW-FQZip 1. Table S4. The performance of Quip (−a). Table S5. The performance of Quip (−r). Table S6. The performance of DSRC 2. Table S7. The performance of CRAM. Table S8. The performance of FQZcomp. Table S9. The performance of LFQC. Table S10. The performance of LEON. Table S11. The performance of SCALCE. Table S12. The performance of bzip 2. Table S13. The performance of gzip. Table S14. The average number of CPU cores used by the compared methods. Table S15. The compression ratios and time consumptions of LW-FQZip 2 w/o complementary palindrome mapping. Table S16. The memory usage of the LW-FQZip 2 w/o complementary palindrome mapping. Table S17. The version information of all compared methods. Table S18. The compression ratios of the compared methods on benchmark data provided by MPEG working group on genomic compression. Table S19. The performance of LW-FQZip 2 on benchmark data provided by MPEG working group on genomic compression. Table S20. The performance of LW-FQZip 2 (−g) on benchmark data provided by MPEG working group on genomic compression. Table S21. The compression ratios of LW-FQZip2 + LCP, LW-FQZip2 -g + LCP, SCALCE, and LW-FQZip 2 on seven representative data sets. Table S22. The comparison of compression speed of LW-FQZip 2 using SSD and HDD disk systems. Figure S1. The compression speeds of LW-FQZip 2 using different number of threads on five representative data sets. Figure S2. The framework of LW-FQZip 2 with LCP technique. (DOCX 229 kb) [file 12859_2017_1588_MOESM1_ESM.docx]

SUPPLEMENT MATERIALS

This document provides the implementation details of LW-FQZip 2 and the detailed experimental results of the comparison studies.

1. **Implementation details of LW-FQZip 2**

LW-FQZip 2 is improved from the LW-FQZip 1 (Zhang, Y., et al. (2015) Light-weight reference-based compression of FASTQ data, BMC bioinformatics, 16, 188.) by introducing more efficient coding scheme and parallelism**.** The detailed procedures of LW-FQZip 2 are provided below in pseudo-code. The main procedure of the program is outlined in Algorithm 1. The compression using PPM prediction model and arithmetic coding is described in Algorithm 2. The source code is available at <http://csse.szu.edu.cn/staff/zhuzx/LWFQZip2>.

| **Algorithm 1**: The main procedure of LW-FQZip 2 | |
| --- | --- |
| **Input:** an FASTQ file ***F*1**, a reference FASTA file ***F*2**, the number of mapping thread ***b***, a set of *k-*mer prefixes ***p***, the length of *k-*mer ***k***, the mismatch tolerance rate ***e***, and the valid repeat length ***l***.  **Output:** an archive file ***F*3**. | |
| **BEGIN** | |
| 1 | Split ***F*1** into ***b*** sub-blocks, |
| 2 | Create ***b*** threads each of which align reads of a corresponding sub-block to ***F*2** using the light-weight mapping model (Zhang et al., 2015) with parameter setting {***p,k,e,l*** }; |
| 3 | Integrate the mapping results (mapped position, palindrome flag, match length, match type and mismatch values) of ***b*** sub-blocks to some intermediate files; |
| 4 | Extract metadata ***M*** and quality scores ***Q*** from ***F*1**; |
| 5 | Encode ***M*** and ***Q*** with incremental coding and run-length-limited coding, respectively (details of the coding schemes are provided in Zhang et al., 2015), in two parallelized threads; |
| 6 | Compress quality scores with PPM prediction model followed by arithmetic coding (see Algorithm 2) or zpaq (<http://mattmahoney.net/dc/zpaq.html>); |
| 7 | Record all intermediate files generated from the metadata and nucleotide sequences by PPM prediction model followed by arithmetic coding or lpaq9m (<http://mattmahoney.net/dc/text.html#1440>), except the mismatch values compacted with improved stationary order arithmetic coding (<http://cs.fit.edu/~mmahoney/compression/text.html#2212>) or zpaq; |
| 8 | Pack the Compression files into ***F*3**; |
| **END** | |

| **Algorithm 2**: Compression with PPM prediction model and arithmetic coding | |
| --- | --- |
| **Input:** an intermediate file ***L***.  **Output:** a coded file ***F****.* | |
| **BEGIN** | |
| 1 | Initialize an ***O***-order context adaptive model ***M*** (***O*** = 0, Maximum: 32) and the probability ranges ***R*** of all symbols in the alphabet; |
| 2 | **For** *i*=1 **to \|*L*\| do // \|*L*\|** is the number of bytes in ***F*** |
| 3 | Read four bytes from ***L*** into a binary string ***C*** ; |
| 4 | **For**  *j*=1 **to** \|***C***\| **do** |
| 5 | Calculate an ***O***-order context probability ***P*** using ***M***; |
| 6 | Calculate new probability ranges of all symbols  ***R’ =R•P***; |
| 7 | **If *M*** predict correctly **then** //update the model ***M***, store the statistics as a count of 1’s and 0’s = *n*_1_/(*n*_0_+*n*_1_) |
| 8 | Increase ***P*** and the number of occurrences of the corresponding ***O***-order context; |
| 9 | Predict the next byte according to model ***M***; |
| 10 | ***O*** = ***O*** + 1; |
| 11 | **Else //**search for the longest and most probably matched context according to the statistics |
| 12 | **While *O* <=** 32 **and** ***O*** > 0 **do** |
| 13 | ***O*** = ***O*** - 8; |
| 14 | **If** the best matched context in ***O*** is detected **then** |
| 15 | ***O*** = ***O*** + 9; |
| 16 | Predict the next byte according to the statistics; |
| 17 | **Break;** |
| 18 | **End If** |
| 19 | **End While** |
| 20 | **End If** |
| 21 | Output the common prefix of ***R’*** to ***F*** and remove the prefix from ***R’***; //e.g., if ***R’ =*** (0.5419…~0.5437…), then ‘54’ is written to ***F*** and ***R’*** becomes (0.19…~ 0.37…); |
| 22 | **End For** |
| 23 | **End For** |
| **END** | |

1. **Detailed experimental results of the comparison studies**

We conducted comparison studies using ten real-world FASTQ files on a platform running 64-bit Red Hat 4.4.7-16 with four 8-core Intel(R) Xeon(R) E7-8837 CPUs (@2.67GHz with Hyper-Threading Technology). LW-FQZip 2, LW-FQZip 2 (-g) is compared to LW-FQZip 1, Quip (-a), Quip (-r), DSRC 2, CRAM, FQZcomp, LFQC, LEON, SCALCE, gzip and bzip 2. All methods are configured to obtain best compression ratios. The detailed results of each method are reported in Tables S1~S13. The average number of CPU cores used by the compared methods are reported in the Table S14. The performance of LW-FQZip 2 with and without complementary palindrome mapping are reported in Tables S15~S16. The version information of all methods used in comparison experiment is shown in the Table S17. The results of the proposed method on benchmark data sets suggested by MPEG working group on genomic compression are provided in Tables S18-S20. Compression ratios of LW-FQZip 2 with LCP technique (framework shown in Fig. S2) are provided in Table S21. The comparison of compression speed of LW-FQZip 2 using SSD and HDD disk systems is presented in Table S22. The compression speeds of LW-FQZip 2 using different number of threads on five representative data sets are plotted in Fig. S1.

**Table S1.** **The performance of LW-FQZip 2 on ten test data sets (command: LWFQZip2 –c –i input.fastq –r reference.fasta)**

|  | Platform | Size(MB) | Compression Ratio | Compression Size(MB) | Compression Time(S) | Decompression Time(S) | Compression mode |
| --- | --- | --- | --- | --- | --- | --- | --- |
| SRR2916693 | 454 GS | 425 | 16.7% | 71 | 35 | 25 | -r NZ_CM002330.1 |
| SRR2994368 | Illumina Miseq | 4688 | 17.3% | 812 | 300 | 240 | -r ecoli |
| SRR3211986 | Pacbio RS | 1759 | 33.3% | 585 | 203 | 400 | -a 0.003 |
| ERR739513 | MinION | 871 | 35.2% | 307 | 122 | 170 | -r BD091641.1 |
| SRR3190692 | Illumina MiSeq | 11379 | 12.7% | 1441 | 540 | 416 | -r ecoli |
| ERR385912 | Illumina Hiseq 2000 | 641 | 6.4% | 41 | 25 | 12 | -r ecoli |
| ERR386131 | Ion Torrent PGM | 1371 | 16.5% | 226 | 87 | 73 | -r NC_000913.3 |
| SRR034509 | Illumina Analyzer II | 5247 | 23.7% | 1241 | 301 | 275 | -r NC_000913.3 |
| ERR174310 | Illumina Hiseq 2000 | 105122 | 21.0% | 22061 | 14050 | 10428 | -r Chr1-4  (Homo sapiens) |
| ERR194147 | Illumina Hiseq 2000 | 202631 | 20.1% | 40812 | 26488 | 19737 | -r Chr1-4  (Homo sapiens) |

**Reference genome ecoli(34MB):NC_000913.3, NC_002695.1, NC_011750.1, NC_011751.1, NC_017634.1, NC_018658.1, AC_000091.1;**

**Table S2. The performance of LW-FQZip 2 (-g)** **on ten test data sets (command: LWFQZip2 –c –i input.fastq –r reference.fasta –g)**

|  | Platform | Size(MB) | Compression Ratio | Compression Size(MB) | Compression Time(S) | Decompression Time(S) | Compression mode |
| --- | --- | --- | --- | --- | --- | --- | --- |
| SRR2916693 | 454 GS | 425 | 15.3% | 65 | 303 | 295 | -r NZ_CM002330.1 |
| SRR2994368 | Illumina Miseq | 4688 | 16.0% | 748 | 1260 | 1198 | -r ecoli |
| SRR3211986 | Pacbio RS | 1759 | 32.3% | 568 | 795 | 725 | -a 0.003 |
| ERR739513 | MinION | 871 | 34.8% | 303 | 333 | 320 | -r BD091641.1 |
| SRR3190692 | Illumina MiSeq | 11379 | 11.7% | 1330 | 2520 | 2372 | -r ecoli |
| ERR385912 | Illumina Hiseq 2000 | 641 | 5.0% | 32 | 282 | 268 | -r ecoli |
| ERR386131 | Ion Torrent PGM | 1371 | 16.0% | 219 | 324 | 301 | -r NC_000913.3 |
| SRR034509 | Illumina Analyzer II | 5247 | 22.7% | 1193 | 1200 | 1080 | -r NC_000913.3 |
| ERR174310 | Illumina Hiseq 2000 | 105122 | 20.1% | 21152 | 42600 | 30000 | -r Chr1-4  (Homo sapiens) |
| ERR194147 | Illumina Hiseq 2000 | 202631 | 14.3% | 28915 | 71400 | 60540 | -r Chr1-4  (Homo sapiens) |

**Table S3. The performance of LW-FQZip 1 on ten test data sets (command: LWFQZip –c –i input.fastq –r reference.fasta)**

|  | Platform | Size(MB) | Compression Ratio | Compression Size(MB) | Compression Time(S) | Decompression Time(S) | Reference |
| --- | --- | --- | --- | --- | --- | --- | --- |
| SRR2916693 | 454 GS | 425 | 18.1% | 77 | 270 | 54 | NZ_CM002330.1 |
| SRR2994368 | Illumina Miseq | 4688 | 17.9% | 841 | 2355 | 742 | ecoli |
| SRR3211986 | Pacbio RS | 1759 | N/A | N/A | N/A | N/A | N/A |
| ERR739513 | MinION | 871 | N/A | N/A | N/A | N/A | N/A |
| SRR3190692 | Illumina MiSeq | 11379 | 13.2% | 1497 | 3945 | 209 | ecoli |
| ERR385912 | Illumina Hiseq 2000 | 641 | 6.6% | 42 | 157 | 52 | ecoli |
| ERR386131 | Ion Torrent PGM | 1371 | 18.7% | 256 | 635 | 148 | NC_000913.3 |
| SRR034509 | Illumina Analyzer II | 5247 | 25.0% | 1313 | 2640 | 285 | NC_000913.3 |
| ERR174310 | Illumina Hiseq 2000 | 105122 | N/A | N/A | N/A | N/A | N/A |
| ERR194147 | Illumina Hiseq 2000 | 202631 | N/A | N/A | N/A | N/A | N/A |

**“N/A”: the program cannot work on the data due to compression program errors;**

**Table S4. The performance of Quip (-a) on ten test data sets (command: quip –a input.fastq –i fastq)**

|  | Platform | Size(MB) | Compression Ratio | Compression Size(MB) | Compression Time(S) | Decompression Time(S) |
| --- | --- | --- | --- | --- | --- | --- |
| SRR2916693 | 454 GS | 425 | 20.9% | 89 | 74 | 29 |
| SRR2994368 | Illumina Miseq | 4688 | 20.1% | 943 | 847 | 637 |
| SRR3211986 | Pacbio RS | 1759 | 33.3% | 585 | 448 | 536 |
| ERR739513 | MinION | 871 | N/A | N/A | N/A | N/A |
| SRR3190692 | Illumina MiSeq | 11379 | 16.5% | 1874 | 2116 | 1329 |
| ERR385912 | Illumina Hiseq 2000 | 641 | 7.2% | 46 | 83 | 88 |
| ERR386131 | Ion Torrent PGM | 1371 | 17.7% | 242 | 84 | 84 |
| SRR034509 | Illumina Analyzer II | 5247 | 25.1% | 1319 | 640 | 522 |
| ERR174310 | Illumina Hiseq 2000 | 105122 | 20.0% | 21042 | 13744 | 6401 |
| ERR194147 | Illumina Hiseq 2000 | 202631 | 20.0% | 40564 | 12398 | 11380 |

**“N/A”: the program cannot work on the data due to compression program errors;**

**Table S5. The performance of Quip (-r) on ten test data sets (command: quip –r reference.fasta input.bam –i bam)**

|  | Platform | Size(MB) | Compression Ratio | Compression Size(MB) | Compression Time(S) | Decompression Time(S) | Reference |
| --- | --- | --- | --- | --- | --- | --- | --- |
| SRR2916693 | 454 GS | 425 | 20.5% | 87 | 81 | 52 | NZ_CM002330.1 |
| SRR2994368 | Illumina Miseq | 4688 | N/A | N/A | N/A | N/A | ecoli |
| SRR3211986 | Pacbio RS | 1759 | N/A | N/A | N/A | N/A | NC_000017.11 |
| ERR739513 | MinION | 871 | N/A | N/A | N/A | N/A | BD091641.1 |
| SRR3190692 | Illumina MiSeq | 11379 | N/A | N/A | N/A | N/A | ecoli |
| ERR385912 | Illumina Hiseq 2000 | 641 | N/A | N/A | N/A | N/A | ecoli |
| ERR386131 | Ion Torrent PGM | 1371 | 16.6% | 228 | 369 | 149 | NC_000913.3 |
| SRR034509 | Illumina Analyzer II | 5247 | 24.9% | 1306 | 3459 | 652 | NC_000913.3 |
| ERR174310 | Illumina Hiseq 2000 | 105122 | N/A | N/A | N/A | N/A | N/A |
| ERR194147 | Illumina Hiseq 2000 | 202631 | N/A | N/A | N/A | N/A | N/A |

**“N/A”: the program cannot work on the data due to compression program errors;**

**Table S6. The performance of DSRC 2 on ten test data sets (command: dsrc2 c –m2 input.fastq output.dsrc)**

|  | Platform | Size(MB) | Compression Ratio | Compression Size(MB) | Compression Time(S) | Decompression Time(S) |
| --- | --- | --- | --- | --- | --- | --- |
| SRR2916693 | 454 GS | 425 | 20.2% | 86 | 20 | 23 |
| SRR2994368 | Illumina Miseq | 4688 | 23.2% | 1087 | 31 | 19 |
| SRR3211986 | Pacbio RS | 1759 | N/A | N/A | N/A | N/A |
| ERR739513 | MinION | 871 | N/A | N/A | N/A | N/A |
| SRR3190692 | Illumina MiSeq | 11379 | 20.3% | 2306 | 37 | 48 |
| ERR385912 | Illumina Hiseq 2000 | 641 | 7.8% | 50 | 12 | 12 |
| ERR386131 | Ion Torrent PGM | 1371 | 16.8% | 230 | 20 | 21 |
| SRR034509 | Illumina Analyzer II | 5247 | 26.1% | 1367 | 110 | 27 |
| ERR174310 | Illumina Hiseq 2000 | 105122 | 20.2% | 21278 | 5450 | 2317 |
| ERR194147 | Illumina Hiseq 2000 | 202631 | 20.3% | 41208 | 4831 | 1800 |

**“N/A”****: the program cannot work on the data due to program core dump;**

**Table S7. The performance of CRAM on ten test data sets (command: java –jar cram.jar cram –I input.bam –O input.cram –R reference.fasta --capture-all-tags -Q)**

|  | Platform | Size(MB) | Compression Ratio | Compression Size(MB) | Compression Time(S) | Decompression Time(S) | Reference |
| --- | --- | --- | --- | --- | --- | --- | --- |
| SRR2916693 | 454 GS | 425 | 21.9% | 93 | 91 | 43 | NZ_CM002330.1 |
| SRR2994368 | Illumina Miseq | 4688 | 26.4% | 1236 | 8411 | 548 | ecoli |
| SRR3211986 | Pacbio RS | 1759 | 33.9% | 597 | 663 | 198 | NC_000017.11 |
| ERR739513 | MinION | 871 | 35.6% | 310 | 227 | 86 | BD091641.1 |
| SRR3190692 | Illumina MiSeq | 11379 | 22.3% | 2541 | 18437 | 1286 | ecoli |
| ERR385912 | Illumina Hiseq 2000 | 641 | N/A | N/A | N/A | N/A | ecoli |
| ERR386131 | Ion Torrent PGM | 1371 | 25.5% | 350 | 303 | 95 | NC_000913.3 |
| SRR034509 | Illumina Analyzer II | 5247 | 27.4% | 1439 | 3196 | 413 | NC_000913.3 |
| ERR174310 | Illumina Hiseq 2000 | 105122 | N/A | N/A | N/A | N/A | N/A |
| ERR194147 | Illumina Hiseq 2000 | 202631 | N/A | N/A | N/A | N/A | N/A |

**“ERR174310”: lose fidelity after decompression;**

**“ERR194147”: the program cannot work on the data due to decompression program errors;**

**“ERR385912”: occurred the compression program errors;**

**Table S8. The performance of FQZcomp on ten test data sets (command: fqz_comp –s9 –q3 input.fastq output.fqz)**

|  | Platform | Size(MB) | Compression Ratio | Compression Size(MB) | Compression Time(S) | Decompression Time(S) |
| --- | --- | --- | --- | --- | --- | --- |
| SRR2916693 | 454 GS | 425 | 21.6% | 92 | 10 | 17 |
| SRR2994368 | Illumina Miseq | 4688 | N/A | N/A | N/A | N/A |
| SRR3211986 | Pacbio RS | 1759 | N/A | N/A | N/A | N/A |
| ERR739513 | MinION | 871 | N/A | N/A | N/A | N/A |
| SRR3190692 | Illumina MiSeq | 11379 | N/A | N/A | N/A | N/A |
| ERR385912 | Illumina Hiseq 2000 | 641 | N/A | N/A | N/A | N/A |
| ERR386131 | Ion Torrent PGM | 1371 | 24.6% | 337 | 34 | 58 |
| SRR034509 | Illumina Analyzer II | 5247 | 26.1% | 1372 | 132 | 216 |
| ERR174310 | Illumina Hiseq 2000 | 105122 | N/A | N/A | N/A | N/A |
| ERR194147 | Illumina Hiseq 2000 | 202631 | N/A | N/A | N/A | N/A |

**“N/A”: lose fidelity after decompression;**

**“ERR739513”: the program cannot work on the data due to decompression program errors;**

**Table S9. The performance of LFQC on ten test data sets (command: ruby lfqc.rb input.fastq)**

|  | Platform | Size(MB) | Compression Ratio | Compression Size(MB) | Compression Time(S) | Decompression Time(S) |
| --- | --- | --- | --- | --- | --- | --- |
| SRR2916693 | 454 GS | 425 | 12.7% | 54 | 286 | 283 |
| SRR2994368 | Illumina Miseq | 4688 | N/A | N/A | N/A | N/A |
| SRR3211986 | Pacbio RS | 1759 | 32.2% | 567 | 1503 | 1493 |
| ERR739513 | MinION | 871 | 34.9% | 303 | 680 | 748 |
| SRR3190692 | Illumina MiSeq | 11379 | N/A | N/A | N/A | N/A |
| ERR385912 | Illumina Hiseq 2000 | 641 | 5.8% | 37 | 644 | 447 |
| ERR386131 | Ion Torrent PGM | 1371 | 15.5% | 213 | 731 | 824 |
| SRR034509 | Illumina Analyzer II | 5247 | 23.7% | 1246 | 3198 | 3138 |
| ERR174310 | Illumina Hiseq 2000 | 105122 | N/A | N/A | N/A | N/A |
| ERR194147 | Illumina Hiseq 2000 | 202631 | N/A | N/A | N/A | N/A |

**“N/A”: the program cannot work on the data due to decompression program errors;**

**Table S10. The performance of LEON on ten test data sets (command: leon –file input.fastq –c -lossless)**

|  | Platform | Size(MB) | Compression Ratio | Compression Size(MB) | Compression Time(S) | Decompression Time(S) |
| --- | --- | --- | --- | --- | --- | --- |
| SRR2916693 | 454 GS | 425 | 19.5% | 83 | 26 | 9 |
| SRR2994368 | Illumina Miseq | 4688 | 23.1% | 1085 | 200 | 48 |
| SRR3211986 | Pacbio RS | 1759 | N/A | N/A | N/A | N/A |
| ERR739513 | MinION | 871 | N/A | N/A | N/A | N/A |
| SRR3190692 | Illumina MiSeq | 11379 | 18.1% | 2057 | 375 | 112 |
| ERR385912 | Illumina Hiseq 2000 | 641 | 7.0% | 45 | 19 | 7 |
| ERR386131 | Ion Torrent PGM | 1371 | N/A | N/A | N/A | N/A |
| SRR034509 | Illumina Analyzer II | 5247 | 27.9% | 1465 | 190 | 44 |
| ERR174310 | Illumina Hiseq 2000 | 105122 | 25.3% | 26560 | 13344 | 1944 |
| ERR194147 | Illumina Hiseq 2000 | 202631 | 20.3% | 41157 | 12273 | 5812 |

**“N/A”: lose fidelity after decompression;**

**Table S11. The performance of SCALCE on ten test data sets (command: scalce-pacbio input.fastq –o inputs)**

|  | Platform | Size(MB) | Compression Ratio | Compression Size(MB) | Compression Time(S) | Decompression Time(S) |
| --- | --- | --- | --- | --- | --- | --- |
| SRR2916693 | 454 GS | 425 | 17.2%# | 73 | 20 | 14 |
| SRR2994368 | Illumina Miseq | 4688 | 17.3%# | 809 | 172 | 93 |
| SRR3211986 | Pacbio RS | 1759 | 33.4%# | 588 | 57 | 29 |
| ERR739513 | MinION | 871 | N/A | N/A | N/A | N/A |
| SRR3190692 | Illumina MiSeq | 11379 | 12.7%# | 1443 | 421 | 207 |
| ERR385912 | Illumina Hiseq 2000 | 641 | 6.6%# | 42 | 27 | 9 |
| ERR386131 | Ion Torrent PGM | 1371 | 16.6%# | 227 | 100 | 24 |
| SRR034509 | Illumina Analyzer II | 5247 | 24.5%# | 1285 | 204 | 82 |
| ERR174310 | Illumina Hiseq 2000 | 105122 | 19.6%# | 20654 | 11379 | 2758 |
| ERR194147 | Illumina Hiseq 2000 | 202631 | 15.4%# | 31105 | 22800 | 4528 |

**“N/A”: the program cannot work on the data due to compression program errors; ‘#’: decompression file listed with no order;**

**Table S12. The performance of bzip 2 on ten test data sets (command: bzip2 –k -9 input.fastq)**

|  | Platform | Size(MB) | Compression Ratio | Compression Size(MB) | Compression Time(S) | Decompression Time(S) |
| --- | --- | --- | --- | --- | --- | --- |
| SRR2916693 | 454 GS | 425 | 24.2% | 103 | 40 | 19 |
| SRR2994368 | Illumina Miseq | 4688 | 28.5% | 1335 | 494 | 233 |
| SRR3211986 | Pacbio RS | 1759 | 36.4% | 641 | 212 | 98 |
| ERR739513 | MinION | 871 | 39.7% | 346 | 109 | 51 |
| SRR3190692 | Illumina MiSeq | 11379 | 24.4% | 2781 | 1097 | 500 |
| ERR385912 | Illumina Hiseq 2000 | 641 | 13.9% | 89 | 104 | 22 |
| ERR386131 | Ion Torrent PGM | 1371 | 21.5% | 295 | 237 | 60 |
| SRR034509 | Illumina Analyzer II | 5247 | 31.5% | 1651 | 598 | 261 |
| ERR174310 | Illumina Hiseq 2000 | 105122 | 26.2% | 27538 | 15794 | 7730 |
| ERR194147 | Illumina Hiseq 2000 | 202631 | 19.7% | 39909 | 21113 | 7362 |

**Table S13. The performance of gzip on ten test data sets (command: gzip -9 input.fastq)**

|  | Platform | Size(MB) | Compression Ratio | Compression Size(MB) | Compression Time(S) | Decompression Time(S) |
| --- | --- | --- | --- | --- | --- | --- |
| SRR2916693 | 454 GS | 425 | 29.6% | 126 | 176 | 7 |
| SRR2994368 | Illumina Miseq | 4688 | 34.2% | 1602 | 2499 | 53 |
| SRR3211986 | Pacbio RS | 1759 | 42.6% | 749 | 725 | 23 |
| ERR739513 | MinION | 871 | 45.4% | 395 | 326 | 13 |
| SRR3190692 | Illumina MiSeq | 11379 | 29.5% | 3357 | 4974 | 122 |
| ERR385912 | Illumina Hiseq 2000 | 641 | 17.9% | 115 | 50 | 5 |
| ERR386131 | Ion Torrent PGM | 1371 | 26.0% | 357 | 145 | 13 |
| SRR034509 | Illumina Analyzer II | 5247 | 36.9% | 1936 | 1567 | 85 |
| ERR174310 | Illumina Hiseq 2000 | 105122 | 31.7% | 33297 | 21132 | 1745 |
| ERR194147 | Illumina Hiseq 2000 | 202631 | 23.6% | 47863 | 26938 | 2007 |

**Table S14. The average number of CPU cores used by the compared methods**

| **Compressor** | **Average CPU cores used** |
| --- | --- |
| **LW-FQZip 2** | 10 |
| **LW-FQZip 2(-g)** | 18 |
| **LW-FQZip 1** | 2 |
| **CRAM** | 27 |
| **FQZcomp** | 1 |
| **DSRC 2** | 24 |
| **Quip(-a)** | 1 |
| **Quip(-r)** | 1 |
| **gzip** | 1 |
| **bzip 2** | 1 |
| **LEON** | 21 |
| **LFQC** | 10 |
| **SCALCE** | 5 |

**Table S15.** **The compression ratios and time consumptions of LW-FQZip 2 with and without complementary palindrome mapping** **on ten test data sets**

|  | LW-FQZip 2  (with the complementary palindrome mapping) | | | | LW-FQZip 2  (without the complementary palindrome mapping) | | | |
| --- | --- | --- | --- | --- | --- | --- | --- | --- |
|  | **Compression Ratio** | **Compression Size(MB)** | **Compression Time(S)** | **Decompression Time(S)** | **Compression Ratio** | **Compression Size(MB)** | **Compression Time(S)** | **Decompression Time(S)** |
| SRR2916693 | 16.5% | 71 | 35 | 25 | 17.6% | 75 | 33 | 22 |
| SRR2994368 | 17.3% | 812 | 300 | 240 | 20.2% | 945 | 390 | 350 |
| SRR3211986 | 33.3% | 585 | 203 | 400 | 33.3% | 585 | 223 | 240 |
| ERR739513 | 35.2% | 307 | 122 | 170 | 35.0% | 305 | 120 | 122 |
| SRR3190692 | 12.7% | 1441 | 540 | 416 | 16.3% | 1859 | 797 | 662 |
| ERR385912 | 6.4% | 41 | 25 | 12 | 6.7% | 43 | 37 | 24 |
| ERR386131 | 16.5% | 226 | 87 | 73 | 16.3% | 224 | 87 | 63 |
| SRR034509 | 23.7% | 1241 | 301 | 275 | 25.3% | 1325 | 536 | 365 |
| ERR174310 | 21.0% | 22061 | 14050 | 10428 | 21.3% | 22329 | 14394 | 10241 |
| ERR194147 | 20.1% | 40812 | 26488 | 19737 | 20.6% | 41757 | 33377 | 15660 |

**Table S16. The memory usage of the LW-FQZip 2 with and without complementary palindrome mapping.**

|  |  | **SRR2916693** | **SRR2994368** | **SRR3211986** | **ERR739513** | **SRR3190692** | **ERR385912** | **ERR386131** | **SRR034509** | **ERR174310** | **ERR194147** |
| --- | --- | --- | --- | --- | --- | --- | --- | --- | --- | --- | --- |
| **LW-FQZip 2**  **(with)** | compression | 1605 | 1582 | 1190 | 1283 | 1726 | 1603 | 1691 | 1748 | 1886 | 1953 |
|  | decompression | 1598 | 1579 | 1528 | 1403 | 1725 | 1603 | 1721 | 1752 | 1865 | 1963 |
| **LW-FQZip 2**  **(without)** | compression | 1544 | 1594 | 1200 | 1287 | 1717 | 1607 | 1662 | 1745 | 1835 | 1899 |
|  | decompression | 1527 | 1563 | 1526 | 1421 | 1713 | 1610 | 1605 | 1652 | 1876 | 1903 |

**Table S17.** **The version information of all compared methods**

|  | **LW-FQZip 1&2** | **Quip** | **DSRC** | **CRAM** | **FQZcomp** | **LFQC** | **LEON** | **SCALCE** | **bzip 2** | **gzip** |
| --- | --- | --- | --- | --- | --- | --- | --- | --- | --- | --- |
| **Version** | 1.02 & 2.0 | 1.1.6 | 2.0 | 3.0 | 4.5 | 1.1 | 1.0.0 | 2.8 | 1.0.6 | 1.6 |

**Table S18. The compression ratios of the compared methods on benchmark data provided by MPEG working group on genomic compression**

**(The data information is available at** <https://github.com/sfu-compbio/compression-benchmark/blob/master/samples.md>**)**

|  | LW-  FQZip 2 | LW-  FQZip 2  (-g) | LW-  FQZip 1 | Quip  (-a) | Quip  (-r) | DSRC 2 | CRAM | FQZcomp | LFQC | LEON | SCALCE | bzip 2 | gzip |
| --- | --- | --- | --- | --- | --- | --- | --- | --- | --- | --- | --- | --- | --- |
| SRR554369 | 20.6% | 19.0% | 21.3% | 20.7% | 22.1% | 23.9% | N/A | N/A | **16.8%** | 20.6% | 19.1%# | 29.7% | 35.6% |
| SRR327342 | 22.9% | **16.9%** | 21.7% | 17.6% | N/A | 22.3% | N/A | N/A | 17.6% | 20.0% | 18.3%# | 28.4% | 33.7% |
| MH0001_081026 | 16.7% | 15.5% | N/A | **14.9%** | N/A | 17.0% | N/A | N/A | 15.4% | 17.2% | 17.2%# | 22.6% | 27.7% |
| SRR1284073 | 32.0% | **31.1%** | N/A | 32.1% | 32.1% | N/A | N/A | N/A | **31.1%** | N/A | 32.2%# | 35.3% | 41.7% |
| SRR870667 | 20.5% | **19.5%** | 22.6% | 20.4% | N/A | 21.4% | 22.8% | N/A | N/A | 24.1% | 18.2%# | 27.3% | 32.1% |

**Note: Because of the lack of appropriate reference, the SRR554369 and SRR327342 are compression by the assemble-based mode (‘-a 0.03’ and ‘-a 0.02’).**

**Table S19. The performance of LW-FQZip 2 (command: LWFQZip2 –c –i input.fastq –r reference.fasta) on benchmark data provided by MPEG working group on genomic compression**

|  | Platform | Size(MB) | Compression Ratio | Compression Size(MB) | Compression Time(S) | Decompression Time(S) | Compression mode |
| --- | --- | --- | --- | --- | --- | --- | --- |
| SRR1284073 | PacBio RS II | 1248 | 32.0% | 399 | 180 | 155 | -r NC_017659.1 |
| SRR554369 | Illumina GAIIx | 733 | 20.6% | 151 | 60 | 49 | -a 0.03 |
| SRR327342 | Illumina GAIIx | 5709 | 22.9% | 1306 | 776 | 566 | -r NC_001133.9 |
| MH0001_081026 | Illumina GAIIx | 3587 | 16.7% | 599 | 288 | 193 | -a 0.02 |
| SRR870667 | Illumina GAIIx | 31693 | 20.5% | 6493 | 2734 | 4380 | -r NC_030850.1 |

**Table S20. The performance of LW-FQZip 2 (-g) (command: LWFQZip2 –c –i input.fastq –r reference.fasta –g) on benchmark data provided by MPEG working group on genomic compression**

|  | Platform | Size(MB) | Compression Ratio | Compression Size(MB) | Compression Time(S) | Decompression Time(S) | Compression mode |
| --- | --- | --- | --- | --- | --- | --- | --- |
| SRR1284073 | PacBio RS II | 1248 | 31.1% | 389 | 612 | 606 | -r NC_017659.1 |
| SRR554369 | Illumina GAIIx | 733 | 19.0% | 139 | 342 | 315 | -a 0.03 |
| SRR327342 | Illumina GAIIx | 5709 | 16.9% | 967 | 1280 | 1164 | -r NC_001133.9 |
| MH0001_081026 | Illumina GAIIx | 3587 | 15.5% | 558 | 781 | 640 | -a 0.02 |
| SRR870667 | Illumina GAIIx | 31693 | 19.5% | 6165 | 6908 | 7375 | -r NC_030850.1 |

**Table S21. The compression ratios of LW-FQZip2+LCP, LW-FQZip2 -g +LCP, SCALCE, and LW-FQZip 2 on seven representative data sets**

|  | Size(MB) | LW-FQZip 2  +LCP | LW-FQZip 2 -g  +LCP | SCALCE | LWFQZip 2 | LWFQZip 2 (-g) |
| --- | --- | --- | --- | --- | --- | --- |
| SRR2916693 | 425 | 14.8%# | 14.8%# | 17.2%# | 16.5% | 15.3% |
| SRR2994368 | 4688 | 16.2%# | 15.6%# | 17.3%# | 17.3% | 16.0% |
| SRR3211986 | 1759 | 33.3%# | 32.8%# | 33.4%# | 33.3% | 32.3% |
| SRR3190692 | 11379 | 11.9%# | 11.4%# | 12.7%# | 12.7% | 11.7% |
| ERR385912 | 641 | 6.2%# | 5.0%# | 6.6%# | 6.4% | 5.0% |
| ERR386131 | 1371 | 16.1%# | 15.8%# | 16.6%# | 16.5% | 16.0% |
| SRR034509 | 5247 | 23.4%# | 22.6%# | 24.5%# | 23.7% | 22.7% |

**Note:** ‘#’: the read order is changed after decompression

**Table S22. The comparison of compression speed of LW-FQZip 2 using SSD and HDD disk systems**

| FASTQ | Size  (MB) | SSD | | | HDD | |
| --- | --- | --- | --- | --- | --- | --- |
|  |  | Compression | Decompression | Compression | | Decompression |
| SRR2916693 | 425 | 16.3MB/s | 22.4MB/s | 6.4MB/s | | 15.7MB/s |
| SRR2994368 | 4688 | 13.5MB/s | 21.0MB/s | 8.5MB/s | | 13.5MB/s |
| SRR3211986 | 1759 | 8.9MB/s | 7.9MB/s | 3.2MB/s | | 5.1MB/s |
| ERR739513 | 8719 | 10.2MB/s | 9.7MB/s | 8.5MB/s | | 9.1MB/s |
| SRR3190692 | 11379 | 14.0MB/s | 17.2MB/s | 9.7MB/s | | 12.7MB/s |
| ERR385912 | 641 | 14.2MB/s | 16.0MB/s | 9.2MB/s | | 11.2MB/s |
| ERR386131 | 1371 | 19.3MB/s | 22.9MB/s | 14.1MB/s | | 15.8MB/s |
| SRR034509 | 5247 | 10.9MB/s | 13.9MB/s | 7.4MB/s | | 10.2MB/s |

**Note:** **We have repeated the experiments on a personal computer (4-core 4.20GHz Intel(R) Core(TM) CPU i7-7700K, single CPU, 16GB RAM) with SSD disks and HDD disks for several FASTQ files.**

**Fig. S1** **The compression speeds of LW-FQZip 2 using different number of threads on five representative data sets**

File sizes: ERR386131 (1.3GB), SRR3190692 (11.1GB), SRR017935 (16.3GB), SRR111960 (69.5GB), ERR174310 (102.7GB).


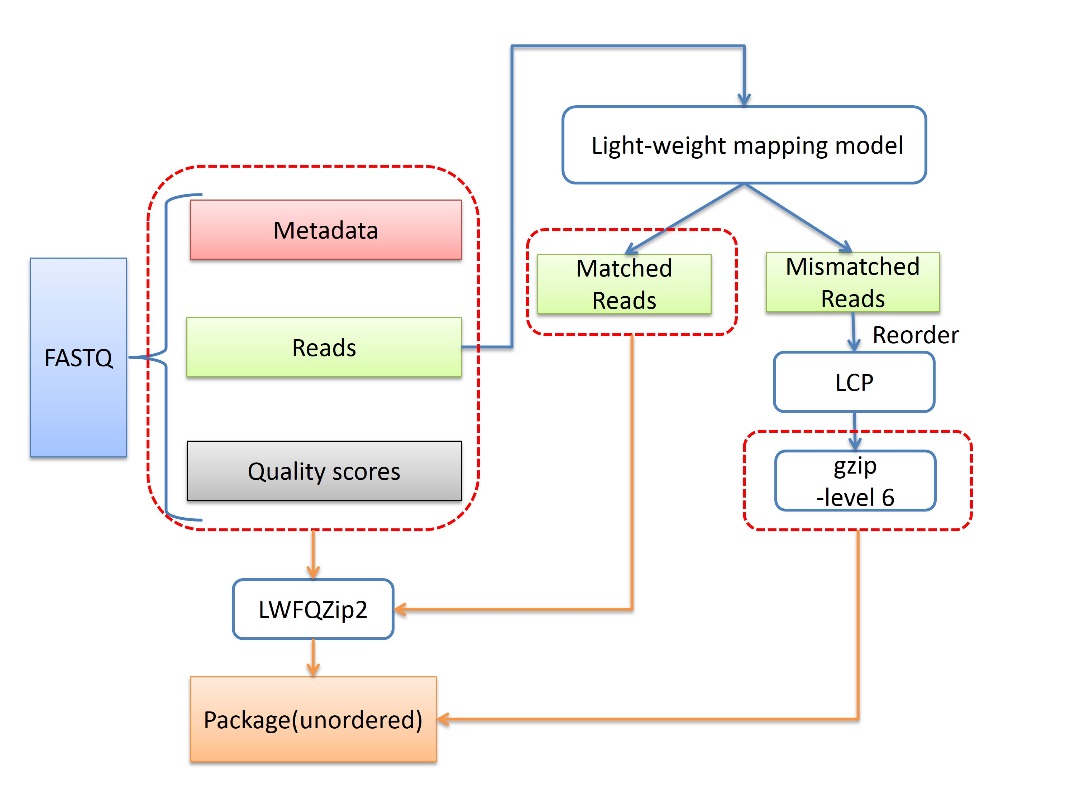


**Fig S2. The framework of LW-FQZip 2 with LCP technique. The successfully mapped reads are compressed with the original LW-FQZip 2, whereas the unmapped reads undergo the LCP boosting and gzip compression.**
